# Large Sample Size Fallacy in Trials About Antipsychotics for Neuropsychiatric Symptoms in Dementia

**DOI:** 10.3389/fphar.2019.01701

**Published:** 2020-02-21

**Authors:** Tessa A. Hulshof, Sytse U. Zuidema, Sarah I. M. Janus, Hendrika J. Luijendijk

**Affiliations:** University Medical Center Groningen, Department of General Practice, University of Groningen, Groningen, Netherlands

**Keywords:** sample size, power, antipsychotics, dementia, placebo-controlled trials, head-to-head trials, meta-epidemiological study

## Abstract

**Background:**

A typical antipsychotics for neuropsychiatric symptoms in dementia have been tested in much larger trials than the older conventional drugs. The advantage of larger sample sizes is that negative findings become less likely and the effect estimates more precise. However, as sample sizes increase, the trials also get more expensive and time consuming while exposing more patients to drugs with unknown safety profiles. Moreover, a large sample size might yield a statistically significant effect that is not necessarily clinically relevant.

**Objective:**

To assess (1) the variation in sample size and sample size calculations of antipsychotic trials in dementia, (2) the size of reported treatment effects and related statistical significance, and (3) general study characteristics that might be related to sample size.

**Study Design and Setting:**

We performed a meta-epidemiological study of randomized trials that tested antipsychotics for neuropsychiatric symptoms in dementia. The trials compared conventional or atypical antipsychotics with placebo or another antipsychotic. Two reviewers independently extracted sample size, sample size calculations, reported treatment effects with p-values, and general study characteristics (drug type, trial duration, type of funding). We calculated a reference sample size of 83 and 433 per study group for the placebo-controlled and head-to-head trials respectively.

**Results:**

We identified 33 placebo-controlled trials, and 18 head-to-head trials. Only 14 (42%) and 2 (11%), respectively, reported a sample size calculation. The average sample size per arm was 34 (range 6–179) in placebo-controlled trials testing conventional drugs, 107 (8–237) in such trials testing atypical drugs, and 104 (95–115) in such trials testing both drug types; it was 31 (10–88) in head-to-head trials. Thirteen out of 18 trials with sample sizes larger than required (72%) reported a statistically significant treatment effect, of which two (15%) were clinically relevant. None of the head-to-head trials reported a statistically significant treatment effect, even though some suggested non-inferiority. In placebo-controlled trials of atypical drugs, longer trial duration (>6 weeks) and commercial funding were associated with higher sample size.

**Conclusion:**

Sample size calculations were poorly reported in antipsychotic trials for dementia. Placebo-controlled trials of atypical antipsychotics showed large sample size fallacy while head-to-head trials were massively underpowered.

## Introduction

Over the years the sample sizes of antipsychotic trials in dementia have increased from as low as 18 in the 1960s to as high as 652 in the 1990s (Schneider et al., 2005; Cox Grad, 2009; Hulshof et al., 2015). The increase in sample sizes is generally viewed as a favorable development. Larger sample sizes provide more power to identify a treatment effect that is really present. In addition, the effect is estimated more precisely (smaller confidence intervals). Larger trials are also a natural consequence of head-to-head trials because the difference between two active drugs is generally expected to be small, and therefore, the required sample size needs to be relatively high.

However, larger sample sizes also make trials expensive and time consuming (Cox Grad, 2009). This can be barrier for non-commercial investigators to perform a trial. Moreover, it can be ethically questionable to ask more patients to participate, especially when the safety of the tested drug has not yet been established (Schipper and Weyzig, 2008). Another disadvantage of (very) large sample size is that a difference in outcomes between the groups will become (very) statistically significant, no matter how small or clinically meaningless it is (Sullivan and Feinn, 2012). If such results are nevertheless interpreted as clinically relevant, the ‘large sample size fallacy’ occurs (Lantz, 2013).

Sample size calculations for trials are based on four parameters if the response rate is the outcome. These are alpha, beta, the expected response rate in the active treatment groups, and the expected response rate in the comparison group (e.g. placebo) (Noordzij et al., 2010). Alpha is the probability of identifying a treatment effect that is not really present, which is usually set at 5%. Beta is the risk of not identifying a treatment effect that is really present, and is usually set at 20%. Sample size calculations for trials with continuous outcomes, such as the reduction of neuropsychiatric symptoms (NPS), are based on alpha, beta, the expected (difference between) means in the active and comparison group, and the population variance around the mean. Furthermore, the expected number of participants dropping out should be taken into account when determining the final target sample size of a trial.

A different expected treatment effect might explain why the sample sizes of antipsychotic trials increased over time. Perhaps, atypical antipsychotics were expected to be less effective than conventional antipsychotics, even before it was shown in systematic reviews that they did not affect psychotic symptoms compared to placebo (Schneider et al., 2006; Smeets et al., 2018). Alternatively, drop-out could have increased because recent trials lasted longer and participants have become more assertive.

On the other hand, general study characteristics, which are not directly related to sample size calculation might have contributed to the increase in trial sample sizes over the years. Large sample size is generally considered a sign of high trial quality, and this increases the probability of publication and citation (Dickersin et al., 1992). In addition, pharmaceutical companies will have more resources to fund larger trials than non-commercial organizations. Therefore, the aim of this meta-epidemiological study was to assess (1) the variation in sample size and sample size calculations of antipsychotic trials in dementia, (2) the size of the reported treatment effects and related statistical significance, and (3) general study characteristics that might be related to sample size.

## Methods

### Search Strategy

Two reviewers (TAH, HJL) used a list of conventional and atypical antipsychotics from the websites of the World Health Organization, Food and Drug Administration, and Wikipedia to search the literature (US Food and Drug Administration, 2013; World Health Organization, 2013; Wikipedia, 2015). First, we first searched for studies in the electronic databases PubMed, CINAHL, EMBASE, and Cochrane library with the string ‘generic name of atypical/conventional antipsychotic’ and trial and dementia (see online supplement). We restricted the position of the drug name to title and abstract. Subsequently, we manually searched the references of published systematic reviews, which were identified with the same electronic databases. Titles and abstracts of potentially eligible studies were retrieved from PubMed. In addition, we sought trials in trial registration websites with the abovementioned search terms if possible; otherwise we used only the term dementia. These three searches were last re-run in June 2019. Finally, we had used the databases of the Dutch Medicines Evaluation Board and the FDA to find unpublished trials as part of a previous search performed in 2015 (Hulshof et al., 2019).

### Study Selection

We screened the title and abstract of the hits. Full texts of potentially eligible published studies and online protocols for unpublished studies were retrieved. Two reviewers used the full texts to determine definitive eligibility (TAH, HJL). The selected trials had to have been randomized and double-blind. They should have tested the efficacy of antipsychotics on NPSs in persons diagnosed with Alzheimer or vascular dementia. The trial had to compare conventional or atypical antipsychotics with placebo or another antipsychotic (head-to-head trial). We excluded studies with multiple drugs in a single intervention arm, studies that were stopped early and thus did not reach the targeted sample size, and studies with a cross-over design as other than standard sample size calculations need to be applied for this design. There were no restrictions with respect to publication date, language, and duration of the study.

### Data Extraction

Two reviewers (TAH or SIMJ and HJL) independently extracted the following general study characteristics besides the sample size from the included studies: placebo-controlled or head-to-head trial, type of dementia (Alzheimer’s disease, vascular dementia, mixed, unspecified), type of NPS (agitation, psychosis, diverse), setting (nursing home, hospital, outpatient clinic), active drug tested (conventional, atypical, or both), trial duration, type of funding (not-for-profit or commercial), and whether a sample size calculation was reported.

If the sample size calculation was reported, we extracted the input for sample size calculations: alpha, beta, expected treatment effects in the comparison groups (response rate, or mean symptom reduction with population variance at endpoint), and the expected drop-out rates. For trials that had been published in an abstract or online trial registration only, this data-extraction was considered inapplicable.

In addition, we extracted the reported treatment effects and related statistical significance. The primary outcome of trials that test antipsychotics for NPS in dementia is most often the difference in response rate or difference in reduction of target symptoms between the treatment groups. We extracted both for each trial with the related p-value. For the response rate, we extracted the number of patients with a clinically relevant improvement as defined by the authors. For reduction in symptoms, we extracted the difference in mean change from baseline to endpoint as measured with a symptom scale, such as the Cohen-Mansfield Agitation Inventory (CMAI) for agitation and Neuropsychiatric Inventory-Nursing Home (NPI-NH) for mixed symptoms. Initially, we also set out to extract standard deviations to calculate standardized mean differences, so that we could compare trial results. However, as many SDs turned out to be missing, we decided to extract the mean on the symptom scale at baseline as a reference instead (see data-analysis).

The primary source of extracted data was the published main results article. If that was not available, then conference abstracts or online published results were used. We received the individual patient data of two trials (Schneider et al., 2006; Paleacu et al., 2008), and additional meta-data of two others for use in another study (De Deyn et al., 1999; De Deyn et al., 2005; Hulshof et al., 2019).

### Data Analyses

First, we described the variation in sample sizes for the different types of trials by plotting the mean number of participants per comparison group against the publication year of the trial. We present these data for the conventional and atypical placebo-controlled trials and head-to head trials separately.

To assess the adequacy of the reported sample sizes, we calculated reference sample sizes for trials with the response rate as outcome. For the placebo-controlled trials, we used an alpha of 0.05, beta of 0.20, a treatment response rate in the antipsychotic group of 55% and in the placebo group of 30%, and an expected drop-out of 30% (Brant, 2016). A treatment effect of 25% (NNT = 4) and drop-out rate of 30% is in line with previous literature and the reported response rates in antipsychotic trials in dementia (Schneider et al., 2006; Jeste et al., 2008; Drouillard et al., 2013). We used a conservative drop-out rate of 30% (it was 26% on average in the included trials), so that the reference sample size would not be an underestimation. The required sample size per study group was 58 without loss to drop-out, and 83 with loss.

For the head-to-head trials (no placebo group), we used a treatment effect of 55% for the drug of interest and 45% for the control antipsychotic drug, because a 10% difference seems the upper limit of no difference. The expected drop-out rate was set at 10%, which is in line with the average drop-out rate in the included head-to-head trials. The required sample size was 389 per group without loss, and 433 with loss. We used the ssi command in Stata version 15.0 to calculate the reference sample sizes (StataCorp, 2017).

To calculate reference sample sizes based on the outcome mean symptom reduction, the minimal clinically important difference (MCID) is required. However, the MCID is not known for most symptom scales used in this field (Shabbir and Sanders, 2014). The exception is the NPI, which was found to have an MCID of at least 8.0 (Howard et al., 2011; Zuidema et al., 2011). Nine of the included placebo-controlled trials in our study used this instrument, and we used the reported data to check our calculated reference sample size based on response rates. The reported mean reduction in symptoms was 19 (SD 14) for the placebo group (see **Supplementary Table 1**), and hence, assuming an MCID of 8.0, 27 (SD 16) for the antipsychotic group. We calculated a required sample size of 80 based on these data, and this finding confirms the reference sample size of 83 based on response rates. In addition, the MCID of 8.0 reflects an SMD of 0.500 given the SD of 16 reported in the included trials. This is in line with the lower limit for a visible (medium) treatment effect suggested by Cohen (2007).

The next step was to assess whether studies with larger sample size reported statistically significant treatment effects that were not clinically relevant (difference in response rate <25%; difference in symptom reduction < MCID or SMD <0.5), which would suggest the presence of large sample size fallacy. Treatment effects in terms of reported response rates can be compared between trials with varying sample sizes. However, it was not possible to use MCIDs or SMDs to compare reported reductions in symptoms across different symptoms scales. Therefore, we calculated the relative symptom reduction as the ratio of the difference in symptom reduction between the study groups relative to the baseline mean in the groups. This approach has been used before (Smith et al., 1974). Moreover, the MCID of 8.0 on the NPI and a mean baseline of 39 (see **Supplementary Table 1**) would translate into a relative symptom reduction of 21%. Hence, a relative symptom reduction of > = 20% seems appropriate.

Finally, we analyzed the association between other general study characteristics and mean sample size per group. The characteristics were type of drug tested (category: conventional, atypical, or both), trial duration (< = 6 weeks, > 6 weeks), and type of funding (non-for-profit, commercial). We calculated mean sample sizes of comparison groups per category, and used the two-sample t-test to determine whether the means differed between the first (reference) category and other categories. The analyses were performed for the placebo-controlled and head-to-head trials separately. All analyses were carried out with Stata version 15.0 (StataCorp, 2017).

## Results

**Figure 1:**
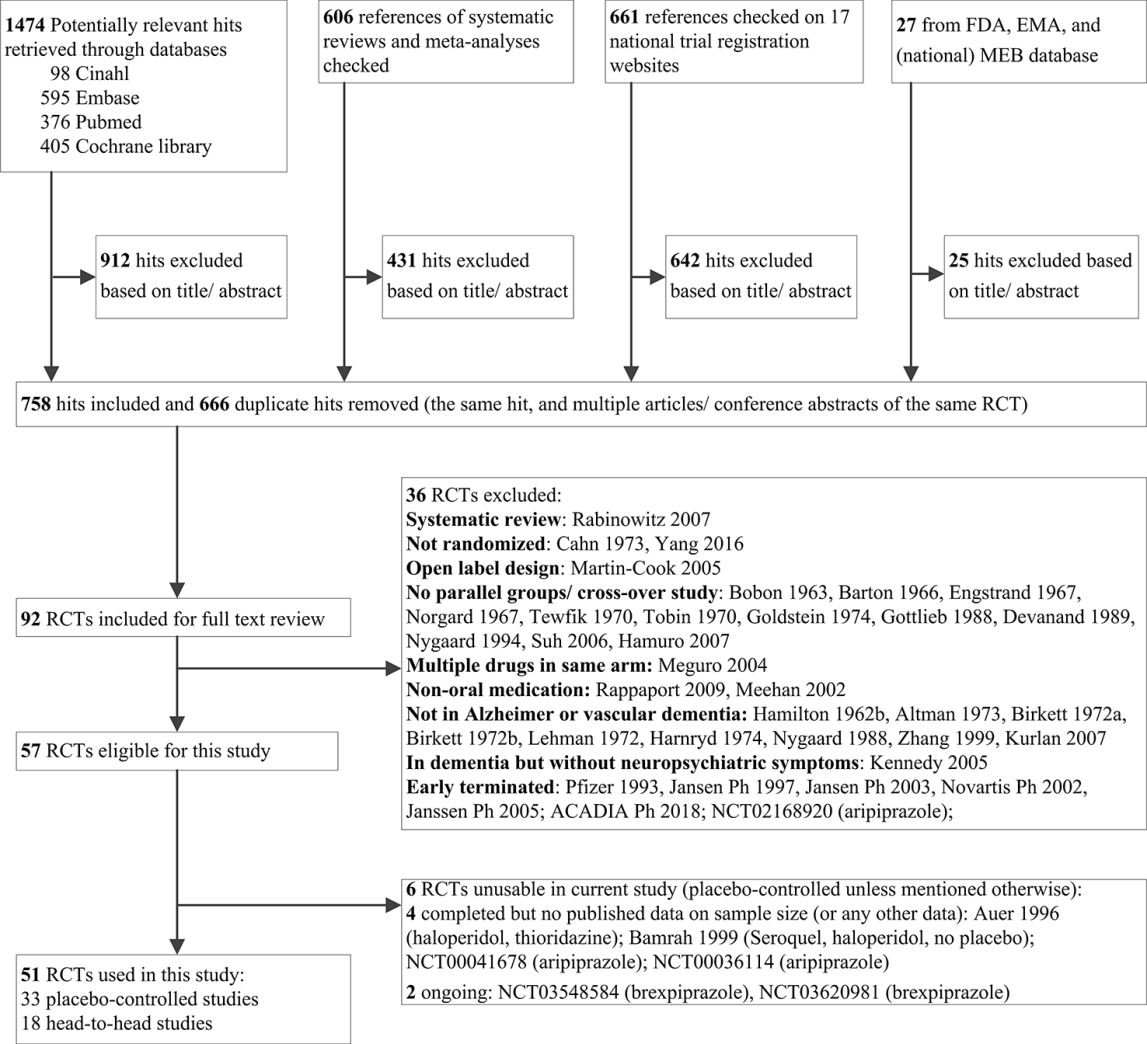
Flow diagram of literature search and study selection.

Our search yielded 2,768 potentially relevant hits (**Figure 1**). We obtained the reports of 92 studies for full text review. We considered 57 studies eligible, but 6 had no useable data at the time of assessment. Hence, we used 51 studies in the current study (Hamilton and Bennet, 1962; Sugerman et al., 1964; Smith et al., 1974; Rada and Kellner, 1976; Rosen, 1979; Vergara et al., 1980; Götestam et al., 1981; Barnes et al., 1982; Petrie et al., 1982; Spagnolo et al., 1983; Morris and Rickels, 1984; Stotsky, 1984; Ather et al., 1986; Lovett et al., 1987; Carlyle et al., 1993; Finkel et al., 1995; Auer et al., 1996; Auchus and Cheryl Bissey-Black, 1997; Devanand et al., 1998; De Deyn et al., 1999; Katz et al., 1999; Allain et al., 2000; Street et al., 2000; Howanitz and Wisotzek, 2001; Herz et al., 2002; Pollock et al., 2002; Brodaty et al., 2003; Fontaine et al., 2003; De Deyn et al., 2004; Garerl et al., 2004; Mulsant et al., 2004; Sheng et al., 2004; Sun et al., 2004; Ballard et al., 2005; De Deyn et al., 2005; Deberdt et al., 2005; Verhey et al., 2006; Tariot et al., 2006; Mintzer et al., 2007; Rainer et al., 2007; Zhong et al., 2007; Paleacu et al., 2008; Streim et al., 2008; Teri et al., 2000). Online or other clinical trial reports of the following studies were used: NCT00287742, NCT01862640, NCT01922258, NCT02992132, ZIP-128-105, RIS-BEL-14, RIS-INT-83.

**Table 1** shows the general study characteristics. Eleven trials compared conventional antipsychotics to placebo and 19 trials atypical antipsychotics to placebo. Six of the latter 19 trials tested multiple doses of one atypical drug, so they had more than one drug group (range 2–4). Three placebo-controlled trials tested both conventional and atypical antipsychotics. Eighteen trials compared an antipsychotic drug with another antipsychotic drug. The studies were performed in outpatients, nursing homes, or hospitals. The target symptom for treatment consisted of agitation, psychosis, or diverse NPSs.

**Table 1:** Characteristics of randomized placebo-controlled and head-to-head trials of antipsychotics in patients with dementia.

Study	Drug(s) studied	Type of dementia	Type of NPS (at least)	Setting	N, total randomized	Duration, weeks	Sample size calculation reported	Commercial funding (drug of sponsor)
***Antipsychotic versus placebo (33)***								
Auchus and Cheryl Bissey-Black, 1997	Haloperidol	AD	Agitation	OUTP	12	6	−	− (non-commercial)
Howanitz and Wisotzek, 2001	Olanzapine	VAS	Diverse NPS	NR	16	6	− (abstract)	NR
Sugerman et al., 1964	Haloperidol	CBS	Psychosis	HOS	18	6	−	+ (haloperidol)
Herz et al., 2002°	Risperidone, olanzapine	AD	Agitation	NR	29	6	− (abstract)	NR
Hamilton and Bennet, 1962	Trifluoperazine	CBS	Psychosis	HOS	27	8	−	NR
Finkel et al., 1995	Thiothixene	NR	Agitation	NH	35	11	−	+ (thiothixene)
Barnes et al., 1982	Loxapine, thioridazine	NR	Diverse NPS	NH	60	8	−	+ (loxapine)
Petrie et al., 1982	Loxapine, haloperidol	NR	Diverse NPS	HOS	63	8	−	+ (loxapine)
Paleacu et al., 2008	Quetiapine	AD	Diverse NPS	NR	40	6	+	+ (quetiapine)
Rada and Kellner, 1976	Thiothixene	CBS	Diverse NPS	HOS	63	4	−	NR
Devanand et al., 1998	Haloperidol	AD	Diverse NPS	OUTP	66	6	−	− (non-commercial)
Ballard et al., 2005	Quetiapine	AD	Agitation	NH	62	6	+	+ (commercial)#
Pollock et al., 2002	Perphenazine	AD, VAS, and MIX	Diverse NPS	NH	54	2,5	−	− (non-commercial)
Teri et al., 2000	Haloperidol	AD	Agitation	HOS	70	16	+	+ (trazodone)
Street et al., 2000	Olanzapine	AD	Diverse NPS	NH	206	6	+	+ (olanzapine)
Ballard et al., 2018	Pimavanserin	AD	Psychosis	NH	181	12*	+	+ (pimvaserin)
Tariot et al., 2006	Quetiapine, haloperidol	AD	Psychosis	NH	284	10	+	+ (quetiapine)
Allain et al., 2000	Tiapride, haloperidol	AD, VAS, and MIX	Agitation	NH-HOS	306	3	+	+ (tiapride)
De Deyn et al., 2005	Aripiprazole	AD	Psychosis	OUTP	208	10	−	+ (aripiprazole)
Zhong et al., 2007	Quetiapine	AD and VAS	Agitation	NH	333	10	+	+ (quetiapine)
Schneider et al., 2006	Olanzapine, quetiapine, risperidone	AD	Diverse NPS	OUTP	421	12^	+	+ (olanzapine, quetiapine, risperidone)
De Deyn et al., 1999	Risperidone, haloperidol	AD, VAS, and MIX	Diverse NPS	NH	344	12	+	+ (risperidone)
Satterlee et al., 1995°	Olanzapine	AD	Diverse NPS	NR	238	8	−	+ (olanzapine)
Mintzer et al., 2007	Aripiprazole	AD	Psychosis	NH	487	10	−	+ (aripiprazole)
Streim et al., 2008	Aripiprazole	AD	Psychosis	NH	265	10	−	+ (aripiprazole)
De Deyn et al., 2004	Olanzapine	AD	Psychosis	NH-HOS	652	10	+	+ (olanzapine)
Otsuka Ph, 2017a†	Brexpiprazole	AD	Agitation	NH	413	12	− (online)	+ (brexpiprazole)
Otsuka Ph, 2017b	Brexpiprazole	AD	Agitation	NH−OUTP	270	12	− (online)	+ (brexpiprazole)
Deberdt et al., 2005	Olanzapine, risperidone	AD, VAS, and MIX	Psychosis	NH−OUTP	494	10	−	+ (olanzapine)
Katz et al., 1999	Risperidone	AD, VAS, and MIX	Diverse NPS	NH	625	12	+	+ (risperidone)
Brodaty et al., 2003	Risperidone	AD, VAS, and MIX	Aggression	NH	345	12	+	+ (risperidone)
Stotsky, 1984	Thioridazine	NR	Diverse NPS	NH-HOS	358	4	−	NR
Mintzer et al., 2006	Risperidone	AD	Psychosis	NH	473	8	+	+ (risperidone)
***Head-to-head trials (18)***								
Vergara et al., 1980	Clomacran vs. thioridazine	CBS	Diverse NPS	HOS	20	12	−	+ (clomacran)
Spagnolo et al., 1983	Clomacran, thioridazine	VAS	Diverse NPS	HOS	30	3	−	NR
Fontaine et al., 2003	Etoperidone, thioridazine	NR	Agitation	NH	39	2	−	+ (olanzapine)
Carlyle et al., 1993	Olanzapine, risperidone	AD, VAS, and MIX	Aggression	HOS	40	4	−	NR
Garerl et al., 2004	Loxapine, haloperidol	AD, VAS, and MIX	Diverse NPS	NR	60	8	−	− (non-commercial)
Morris and Rickels, 1984	Risperidone, olanzapine, promazine	NR	Diverse NPS	NH	41	8	−	+ (loxapine)
Rosen, 1979	Loxapine, thioridazine	Organic cerebral disease#	Diverse NPS	OUTP	56	6	−	+ (haloperidol)
Smith et al., 1974	Haloperidol, thioridazine	CBS	Psychosis	NH	46	6	−	NR
Götestam et al., 1981	Haloperidol, thioridazine	(Pre)senile and VAS	Diverse NPS	HOS	47	8	−	NR
Lovett et al., 1987	Cis(Z)−clopenthixol, haloperidol	CBS	Psychosis	NH	54	6	−	+ (trifluoperazine)
Chan et al., 2001	Trifluoperazine, haloperidol	AD, VAS, and MIX	Diverse NPS	OUTP−HOS	58	12	−	+ (risperidone)
Verhey et al., 2006	Risperidone, haloperidol	NR	Agitation	OUTP-NH	59	5	+	NR
Ather et al., 1986	Olanzapine, haloperidol	NR	Diverse NPS	NR	68	4	−	+ (chlormethiazole)
Sheng et al., 2004	Chlormethiazole, thioridazine	AD and VAS	Diverse NPS	NR	60	8	−	+ (risperidone)
Rainer et al., 2007	Risperidone, haloperidol	AD, VAS, MIX, FTD	Diverse NPS	OUTP	68	8	+	+ (quetiapine)
Mulsant et al., 2004	Quetiapine, risperidone	AD, VAS and MIX	Diverse NPS	NH	86	6	−	+ (risperidone)
Sun et al., 2004	Risperidone, olanzapine	DSM-IV dementia	Diverse NPS	HOS-OUTP	116	8	−	+ (risperidone)
Gutzmann et al., 1997	Risperidone, haloperidol	NR	Restlessness	HOS	176	4	−	+ (tiapride)

AD stands for Alzheimer’s disease; CBS for chronic brain syndrome; HOS for hospital; MIX for mixed dementia (Alzheimer/vascular); NH for nursing home; NPS for neuropsychiatric symptoms; OUTP for outpatients; Ph for Pharmaceutical company; NR for not reported; and VAS for vascular dementia.

° abstract only; * reduction in NPI Psychosis items at 12 weeks was the original primary outcome (clinicaltrials.gov); ^ discontinuation rate at week 36 was the primary outcome, but as it is incomparable to other trials, we used response rate and reduction of symptoms at 12 weeks (see **Table 3**); † results of 0.5 mg group (n = 20) were not reported; # the term senile brain disease was also used.

### Sample Size Variation and Calculations

**Figure 2:**
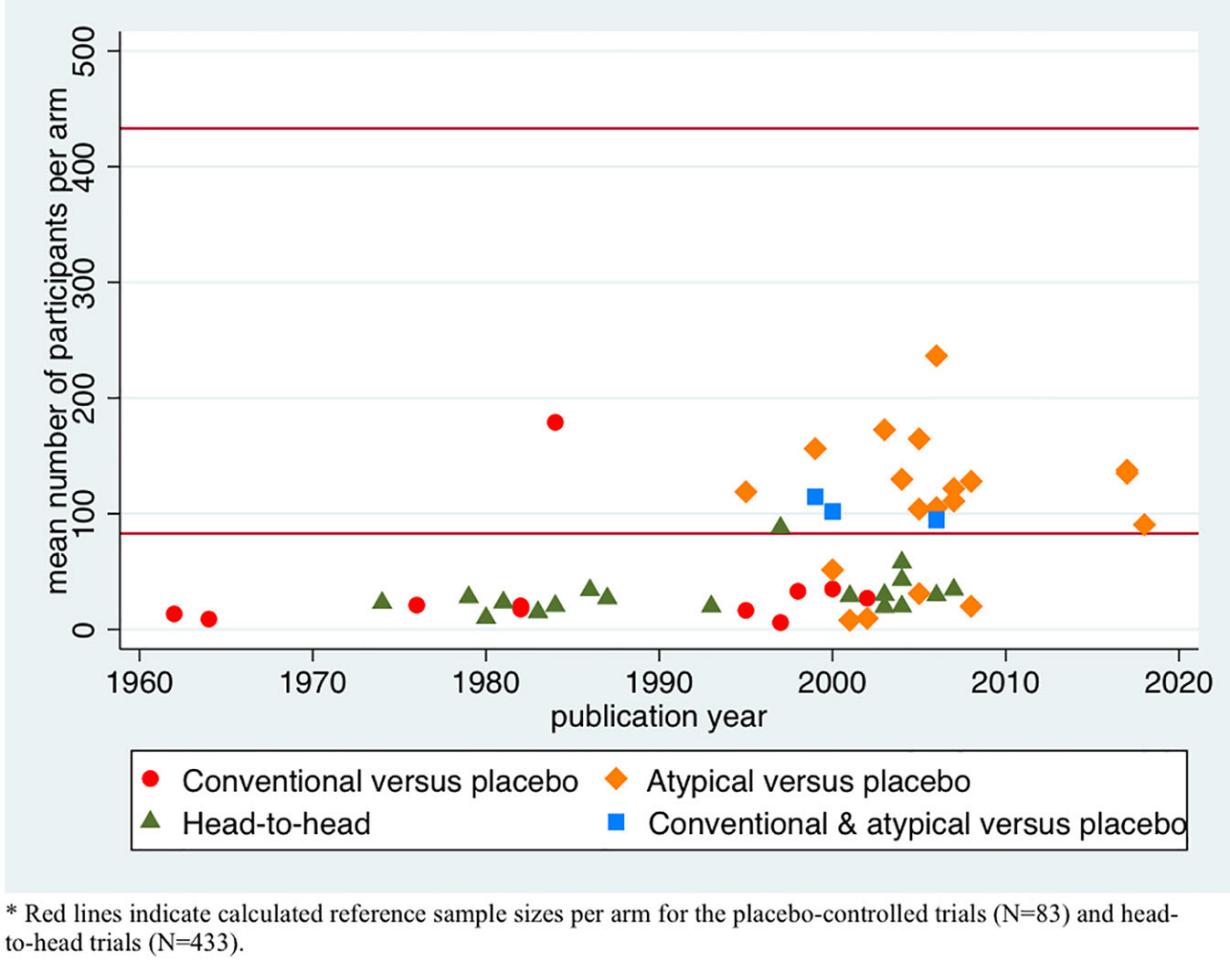
Scatter plot of sample sizes per arm over the years per treatment group.

**Figure 2** shows the mean number of participants per comparison group in each trial against publication year. The symbols indicate the type of drug tested (conventional, atypical, or both) and type of study (placebo-controlled or head-to-head). In the conventional antipsychotic placebo-controlled studies, the mean number per group was 34 patients (range 6–179), while those comparing atypical antipsychotics to placebo included on average 107 patients per group (range 8–237). The three trials that included both conventional and atypical antipsychotics and compared these to placebo included 104 patients per group (range 95–115). Head-to-head trials included a mean number of 31 patients per group (range 10–88). The increase in sample size over time seems to be related to type of drug tested.

We calculated a reference sample size of 83 patients per group for the placebo-controlled trials and 433 patients for the head-to-head trials, as explained above. The group sample size was lower than the reference sample size in 10 placebo-controlled trials of conventional antipsychotics (small sample size) and higher in one such trial (large sample size), whereas 5 of the 19 atypical antipsychotic trials and none of the 3 trials including both conventional and atypical antipsychotics had small sample sizes. At least four of the five atypical underpowered antipsychotic trials were investigator initiated, although one was performed with commercially acquired funds. All head-to-head trials had a small sample size that was lower than the reference sample size of 433.

Sixteen of 47 articles (excluding 2 abstracts and 2 reports on online trial registers) reported a sample size calculation (34%), which was often called a power analysis (**Table 1**). Fourteen were placebo-controlled trials and two head-to-head trials (**Table 2**). **Table 2** shows, which input for these sample size calculations was reported. There were only three studies that reported sufficient information (Ballard et al., 2005; Mintzer et al., 2006; Schneider et al., 2006). Two studies reported an alpha that differed from 5% (2.5% and 7%). Eight studies reported a beta that differed from 20% and it varied between 1% and 15%. Except for the alpha of 2.5%, this input will yield higher sample sizes. Expected drop-out rates were reported in seven studies and varied between 10% and 30%.

**Table 2:** Input for sample size calculations*.

Study	Alpha, %	Beta, %	Response rate or mean symptom change in drug group	Response rate or mean symptom change in control group	Difference in rates or means (SD) between groups¶	Expected dropout, %
**Placebo-controlled trials**						
Teri et al., 2000†	5	20	70%	30%	40%	NR
Katz et al., 1999	5	20	50%	30%	20%	NR
Street et al., 2000	5	20	NA	NA	−2.0 pts (NR)	NR
Brodaty et al., 2003	5	20	NA	NA	−4.15 pts (NR)	30
De Deyn et al., 2004	5	15	NA	NA	−3.0 pts (NR)	NR
Ballard et al., 2005	5	10	NA	NA	−6.0 pts (6)	25
Schneider et al., 2006	5	1^	27%#	60%#	−33%#	NA#
Mintzer et al., 2006	5	5	45%	25%	20%	20
Zhong et al., 2007	2.5	20	NR	NR	NR	10
Paleacu et al., 2008	7	10	NR	NR	−25% pts (NR)	NR
Ballard et al., 2018	5	10	NA	NA	−3.0 (6)	20
De Deyn et al., 1999	5	20	NR	NR	20%	20
Allain et al., 2000	5	20	55%	30%	25%	NR
Tariot et al., 2006	5	10	NA	NA	−4.5 (9)	NR
**Head-to-head trials**						
Verhey et al., 2006	5	10$	−14 pts	−2.8 pts	<−11.2 (NR)	25
Rainer et al., 2007	5	20	NR	NR	NR	NR

NA stands for not applicable, NR for not reported, pts for points (on instrument used to measure neuropsychiatric symptoms); * this table presents the 16 studies that reported a sample size calculation (‘power analysis’) were included in this table; ¶ a difference in means needs to be accompanied by the population variance to calculate a sample size; † except for Teri et al., 2000, all calculations were based on the comparison of the atypical antipsychotic group versus placebo; ^ beta was reported to be 20% for a difference in rates of -20%; # discontinuation (not response) was the outcome; $ text also mentions 20%.

There were seven placebo-controlled trials that postulated an expected treatment effect in terms of symptom reduction, four of which reflected a relative symptom reduction below 20%. The expected differences in relation to baseline means (relative symptom reduction) were: 10% (Ballard et al., 2005); 11% (Tariot et al., 2006); 12% (Brodaty et al., 2003); 14% (Street et al, 2000); 20% (Mintzer et al., 2006); 31% (De Deyn et al., 2004); 31% (Ballard et al., 2018). For a head-to-head trial, the expected relative risk reduction was 16% (Verhey et al., 2006).

### Reported Treatment Effects In Relation To Sample Size

**Table 3** presents the reported treatment effects in order of sample size per study group. A positive difference in response rate and negative difference in symptom reduction means that the investigated drug performed better than the control group. Six trials did not report what the effect of treatment on the primary outcome was: four studies were old, published between 1974–1983, but two were relatively new, published after 2000 (Smith et al., 1974; Rada and Kellner, 1976; Vergara et al., 1980; Spagnolo et al., 1983; Herz et al., 2002; Mulsant et al., 2004). Five placebo-controlled studies reported only p-values without effect sizes in the abstract (Katz et al., 1999; Brodaty et al., 2003; Deberdt et al., 2005; Mintzer et al., 2006; Zhong et al., 2007).

**Table 3:** Results of randomized trials in order of group sample size.

Study	Comparison groups	N per group	Reported effect in terms of response rate			Reported effect in terms of symptom reduction		
			Definition/measurement (bold if primary outcome)	Difference between groups	p-value	Symptom scale (bold if primary outcome)	Difference between groups (baseline mean); relative symptom reduction	p-value
***Antipsychotic versus placebo (33)***								
Auchus and Cheryl Bissey-Black, 1997	Haloperidol vs. placebo	6–6	—	—	—	**CMAI**	−1.0 (35.2); 5%	.82
Howanitz and Wisotzek, 2001	Olanzapine vs. placebo	8–8	—	—	—	—	—	—
Sugerman et al., 1964	Haloperidol vs. placebo	9–9	**improvement on psychiatric observation**	**22%**	nr	‘symptom checklist’	−2.5 (nr); nr	nr
Herz et al., 2002°	Risperidone vs. placebo	14–8	—	—	—	**BPRS Excitement**	Nr (nr); nr	ns .0001
	Olanzapine vs. placebo	7–8	—	—	—		Nr (nr); nr	
Hamilton and Bennet, 1962	Trifluoperazine vs. placebo	18–9	**improvement on psychiatric observation**	**22%**	nr	MACC	−0.7 (31.4); 2%	ns
Finkel et al., 1995	Thiothixene vs. placebo	17–18	> 5 points on CMAI	51%	nr	**CMAI**	−9.0 (30.5); 55%	<.001
Barnes et al., 1982	Loxapine vs. placebo Thioridazine vs. placebo	19–17 17–17	improvement on CGI	17% 12%	ns ns	**BPRS**	−2.9 (45.8); 6% 0.0 (45.8); 0%	ns ns
Petrie et al., 1982	Loxapine vs. placebo Haloperidol vs. placebo	19–22 20–22	**> = moderate improvement on CGI**	23% 26%	nr nr	BPRS	−9.5 (47.9); 20% −9.3 (47.9); 19%	<.05 <.05
Paleacu et al., 2008	Quetiapine vs. placebo	20–20	Improved on CGIC	−5%	ns	**NPI-NH**	−5.2 (41.0); 13%	ns
Rada and Kellner, 1976	Thiothixene vs. placebo	22–20	improved on global rating	4%	ns	**BPRS**	Nr (nr); nr	ns
Devanand et al., 1998	Haloperidol 0.5–0.75 mg vs. placebo Haloperidol 2–3 mg vs. placebo	21–24 21–24	> = 25% reduction BPRS Psychosis items	0% 30%	nr <0.06	**BPRS Psychosis**	0.0 (6.8); 0% −1.2 (6.8); 18%	ns <.03
Ballard et al., 2005	Quetiapine vs. placebo	31–31	—	—	—	**CMAI**	3.5 (57.7); 8%	.30
Pollock et al., 2002	Perphenazine vs. placebo	33–21	—	—	—	**NRS**	−4.9 (57.6); 9%	.14
Teri et al., 2000	Haloperidol vs. placebo	34–36	**improvement on ADCS-CGIC**$	**1%**	0.81	CMAI	−1.3 (49.2*); 3%	>.25
Street et al., 2000	Olanzapine 5 mg vs. placebo Olanzapine 10 mg vs. placebo Olanzapine 15 mg vs. placebo	56–47 50–47 53–47	— — —	— — —	— — —	**NPI-NH Agitation + Psychosis**	−3.9 (14.2); 27% −2.4 (14.2); 17% −1.2 (14.2); 8%	<.001 .006 .24
Ballard et al., 2018¶	Pimavanserin vs. placebo	90–91	> = 30% decrease on NPI-NH Psychosis items	nr	nr	**NPI-NH Psychosis**	−0.5 (9.8); 5%	.561
Tariot et al., 2006	Quetiapine vs. placebo Haloperidol vs. placebo	91–99 94–99	> = 30% decrease on BPRS	11% 7%	.265 nr	BPRS	−2.3 (39.5); 6% −0.4 (39.5); 1%	.217 .354
Allain et al., 2000	Tiapride vs. placebo Haloperidol vs. placebo	102–103 101–103	**> = 25% decrease on MOSES irritability/aggression items**)	14% 20%	.04 .004	MOSES irritability/aggression	−1.9 (20.3); 9% −2.1 (20.3); 10%	.009 .005
Deberdt et al., 2005	Aripiprazole vs. placebo	106–102	improvement on CGI-I	8%	.18	**NPI Psychosis**	−1.03 (12.4); 8%	.017
Zhong et al., 2007	Quetiapine 100 mg vs. placebo Quetiapine 200 mg vs. placebo	124–92 117–92	moderate and marked improvement on CGI-C	8% 22%	ns .002	**PANSS-EC**	−0.8 (23.0); 3% −2.7 (23.0); 12%	.457 .014
Schneider et al., 2006	Olanzapine vs. placebo Quetiapine vs. placebo Risperidone vs. placebo	100–142 94–142 85–142	improvement on CGIC†	11% 5% 8%	.05 .37 .21	NPI	−5.0 (36.9); 14% −7.6 (36.9); 21% −7.4 (36.9); 20%	nr nr nr
De Deyn et al., 1999	Risperidone vs. placebo Haloperidol vs. placebo	115–114 115–114	**> = 30% decrease on BEHAVE-AD**	11% 8%	.13 .25	BEHAVE-AD BEHAVE-AD	−2.4 (16.5); 15% −1.3 (16.5); 8%	.05 nr
Satterlee et al., 1995°	Olanzapine vs. placebo	120–118	—	—	—	BEHAVE-AD	−0.4 (19.8); 2%	ns
Mintzer et al., 2007	Aripiprazole 2 mg vs. placebo Aripiprazole 5 mg vs. placebo Aripiprazole 10 mg vs. placebo	118 –121 122–121 126–121	> = 50% decrease NPI-NH Psychosis	5% 13% 15%	ns ns .019	**NPI-NH Psychosis**	−0.5 (11.6); 4% −1.2 (11.6); 10% −1.8 (11.6); 16%	ns ns .013
Streim et al., 2008	Aripiprazole vs. placebo	131–125	> = 50% decr NPI-NH	18%	.006	**NPI-NH Psychosis**	+0.1 (10.6); 1%	ns
De Deyn et al., 2004	Olanzapine 1mg vs. placebo Olanzapine 2.5 mg vs. placebo Olanzapine 5 mg vs. placebo Olanzapine 7.5 mg vs. placebo	129–129 134–129 125–129 132–129	— (CGI—C was administered)	— — — —	— — — —	**NPI**—**NH Psychosis**	−1.0 (9.7); 10% −0.8 (9.7); 8% −0.6 (9.7); 6% −1.2 (9.7); 12%	.171 .089 .274 .032
Otsuka Ph, 2017a^	Brexpiprazole 1 mg vs. placebo Brexpiprazole 2 mg vs. placebo	137–136 140–136	—	—	—	**CMAI**	+0.2 (nr); nr −3.8 (nr); nr	.902 .040
Otsuka Ph, 2017b	Brexpiprazole vs. placebo	133–137	—	—	—	**CMAI**	−2.4 (nr); nr	.145
Deberdt et al., 2005	Olanzapine vs. placebo Risperidone vs. placebo	204–94 196–94	> = 30% decr NPI-NH Psychosis	−4% −3%	ns ns	**NPI Psychosis**	−0.7 (11.3); 6% −0.5 (11.3); 4%	0.421 0.585
Katz et al., 1999	Risperidone 0.5 mg vs. placebo Risperidone 1 mg vs. placebo Risperidone 2 mg vs. placebo	149–163 148–163 165–163	> = 50% reduction on BEHAVE-AD	nr 12% 17%	nr .02 .002	**BEHAVE-AD**	−1.2 (15.8); 8% −2.2 (15.8); 14% −3.3 (15.8); 21%	.13 .02 <.001
Brodaty et al., 2003	Risperidone vs. placebo	173–172	improvement on CGI-I	22%	< .001	**CMAI aggression**	−4.4 (33.5); 23%	<.001
Stotsky, 1984	Thioridazine vs. placebo	183–175	—	—	—	**Modified HAS**	−4.3 (nr); nr	<.001
Mintzer et al., 2006	Risperidone vs. placebo	235–238	improvement on CGI-C	10%	.019	**BEHAVE-AD Psychosis**	−0.6 (7.9); 8%	.118
***Head-to-head trials (18)***								
Vergara et al., 1980	Clomacran vs. thioridazine	20 total	Improvement on CGI	0%	nr	**VTSRS**	nr (nr); nr	ns
Spagnolo et al., 1983	Etoperidone vs. thioridazine	15–15	clinical judgment	0%	nr	**SHGRS**	nr (nr); nr	nr
Fontaine et al., 2003	Olanzapine vs. risperidone	20–19	— (CGI-C was administered)	nr	ns	**NPI**	+8 (51.8); 15%	ns
Carlyle et al., 1993	Loxapine vs. haloperidol	20–20	Any decrease in weekly # of aggressive acts	15%	nr	**weekly # of aggressive acts**	−1.1 (6.9); 16%	ns
Garerl et al., 2004	Risperidone vs. promazine Olanzapine vs. promazine	20–20 20–20	**> = 50% decrease on NPI**	5% 15%	nr nr		— —	— —
Morris and Rickels, 1984	Loxapine vs. thioridazine	21–20	global improvement	nr	nr	**BPRS**	+1.7 (63.6); 3%	ns
Rosen, 1979	Haloperidol vs. thioridazine	24–18	—	—	—	**Modified BPRS**	+0.1 (3.2); 3%	ns
Smith et al., 1974	Haloperidol vs. thioridazine	23–23	CGI	22%	nr	**BPRS**	nr (nr); 11%	.01
Götestam et al., 1981	Cis(Z)-clopenthixol vs. haloperidol	25–22	improvement on CGI	−6%	nr	**GCGRS**	−4.1 (26.9); 15%	<.05
Lovett et al., 1987	Trifluoperazine vs. haloperidol	26–28	improvement on CGI	18%	ns	**BPRS**	−1.2 (50.4); 2%	ns
Chan et al., 2001	Risperidone vs. haloperidol	29–29	—	—	—	**CMAI**	+2.0 (47.7); 4%	ns
Verhey et al., 2006	Olanzapine vs. haloperidol	30–28	— (CGI was administered)	—	—	**CMAI**	+6.5 (70); 9%	0.338
Ather et al., 1986	Chlormethiazole vs. thioridazine	30–30	—	—	—	**CGBRS**	−1.9 (37.1); 5%	nr
Sheng et al., 2004	Risperidone vs. haloperidol	30–30	**improvement on CGI**	10%	>.05	**BEHAVE-AD**	0 (15); 0%	>.05
Rainer et al., 2007	Quetiapine vs. risperidone	36–32	improvement on CGI	−3.4%	nr	**NPI**	+2.2 (57.9); 4%	ns
Mulsant et al., 2004	Risperidone vs. olanzapine	42–43	—	—	—	**NPI**	Nr (nr); nr	ns
Sun et al., 2004	Risperidone vs. haloperidol	57–59	**> = 30% decrease on BEHAVE-AD**	1%	nr	**BEHAVE-AD**	+0.1 (17.5); 1%	ns
Gutzmann et al., 1997	Tiapride vs. melperone	88–87	**improvement on CGI**	1%	.675	restlessness	−1.4 (56.2); 2%	ns

nr stands for not reported, ns means that the effect was reported as not statistically significant but no p-value was given; ADCS-CGIC stands for Alzheimer’s Disease Cooperative Study Clinical Global Impression of Change; BEHAVE-AD for Behavioural pathology in Alzheimer’s disease scale; BPRS for Brief Psychiatric Rating Scale; CMAI for Cohen-Mansfield Agitation Inventory; CGBRS for Crichton Geriatric Behavioral Rating Scale; GCGRS for Gottfires-Cronholm Geriatric Rating Scale; MACC for Motility affect communication cooperation behavioral adjustment scale; NPI (-NH) for Neuropsychiatric Inventory (- Nursing Home version); NRS for Neurobehavioral Rating Scale; PANSS for Positive and Negative Syndrome Scale; SHGRS for Stuard Hospital Geriatric Rating Scale; and VTSRS for Verdun Target Symptom Rating Scale.

° abstract only; * 49.2 is the weighted mean of baseline mean of all studies with CMAI total; ¶ reduction in NPI Psychosis at 12 weeks was originally the primary outcome (clinicaltrials.gov); † Discontinuation rate at week 36 is primary outcome of trial, but as it is incomparable to other trials, we used response rate and reduction of symptoms at 12 weeks; ^ results of 0.5mg group (n = 20) were not reported.

Thirteen of 18 overpowered trials (72%) versus seven of 15 underpowered placebo-controlled trials (47%) yielded a statistically significant difference between the study groups in either response rate or symptom reduction. Two of 13 (15%) and four of seven (57%) of these treatment effects respectively were clinically relevant (difference in response rate > = 25%, or relative symptom reduction > = 20%). The statistically significant response rates were 10–22% and reported by studies with large sample sizes. The two studies with a difference in response rate of > = 25%, which is the difference deemed clinically relevant (Cohen, 2007), were underpowered and did not report a statistically significant result. In addition, large sample size trials reported statistically significant relative symptom reductions between 10% and 23%, and small sample size trials reported statistically significant relative symptom reductions varying between 17% and 55%.

Many placebo-controlled trials had more than one intervention group, adding up to a total of 54 individual comparisons. Thirteen of the 33 overpowered comparisons (39%) from 18 trials yielded a statistically significant treatment effect on either response rate or symptom reduction, versus seven of the 21 underpowered comparisons (33%) from 15 trials.

Five of 18 head-to-head trials reported a difference in response rate of 10%, the lower limit that we set for non-inferiority in our reference sample size calculation, and four a relative symptom reduction of 10%. Yet, none of these results were statistically significant.

The reported treatment effect was lower than the expected treatment effect in the 14 studies that presented an expected treatment effect in a sample size calculation, except in two studies (Street et al., 2000; Brodaty et al., 2003). The reported drop-out rates varied between 6% and 37% (not shown), which was higher than the expected drop-out rate in most studies.

### Study Characteristics and Sample Size

**Table 4** shows the mean sample size per comparison group by type of drug tested, trial duration, and type of funding. The mean sample size per study group was statistically significantly higher in placebo-controlled trials that tested an atypical antipsychotic drug (107.0) or both a conventional and an atypical drug (103.8) in comparison to placebo-controlled trials of conventional antipsychotics (34.4; p < .05). The mean sample size per study group was also statistically significantly higher in trials that lasted more than 6 weeks (109.2) compared to less than 6 weeks (28.9; p < .001), and that were commercially (100.3) versus non-commercially (18.1; p < .001) funded. Head-to-head-trials that tested atypical drugs only had a significantly larger mean sample size (46.3) than trials that tested conventional drugs (22.3; p < .05). Trial duration and commercial funding did not seem to be related to the sample size of head-to-head trials.

**Table 4:** Mean sample size by study characteristic.

Study characteristic		Placebo-controlled trials		Head-to-head trials	
		n	Mean (SD)	n	Mean (SD)
Type of drug	Conventional antipsychotic (ref)	11	34.4 (48.8)	9	22.3 (7.1)
	Atypical antipsychotic	19	107.0 (60.5)^	4	46.3 (29.5)^
	Conventional and atypical antipsychotic	3	103.8 (94.7)^	3	33.3 (14.4)
Trial duration	= < 6 weeks (ref)	11	28.9 (4.4)	10	32.7 (21.0)
	> 6 weeks	22	109.2 (12.6)*	8	28.2 (14.2)
Type of funding	Non-commercial (ref)	7	18.1 (9.6)	5	21.6 (5.4)
	Commercial	24	100.3 (57.5)*	13	34.2 (20.0)

^p < .05 compared to reference group; *p < .001 compared to reference group.

## Discussion

We assessed the presence of large sample size fallacy in 51 antipsychotic trials in dementia. Most placebo-controlled trials of conventional antipsychotics had small sample size, i.e. smaller than the calculated reference sample size, but most trials of atypical antipsychotics had large sample sizes. All head-to-head trials had very small sample sizes. Only one third of trials reported a sample size calculation. Thirteen of 18 trials with large sample sizes (72%) reported a statistically significant treatment effect, of which two (15%) were clinically relevant. In contrast, seven of 15 placebo-controlled trials with small sample sizes (47%) yielded a statistically significant treatment effect, and four were clinically relevant (57%). None of the head-to-head trials reported a statistically significant treatment effect, even though some suggested non-inferiority.

### Large Sample Size Fallacy

Sample sizes need to be large enough to guarantee a minimum level of discriminative power to detect a real treatment effect. Moreover, precision of an estimate increases with sample size. Studies based on small sample size may yield a non-statistically significant but clinically relevant treatment effect. On the other hand, studies based on large sample size—larger than necessary—may yield statistically significant but clinically insignificant treatment effects (Roggla and Fortunat, 2004; Chan et al., 2008). Large sample size fallacy occurs when such results are interpreted as relevant for medical practice (Lantz, 2013; Lin et al., 2013). Nevertheless, pharmaceutical companies and academic scholars benefit from statistically significant treatment results being interpreted as clinically relevant (Dickersin et al., 1992). The emphasis on statistical significance was confirmed by six trials in our review that did not report effect sizes, and five trials that reported just p-values in the abstract.

The sample sizes of trials testing atypical antipsychotics versus placebo, whether or not simultaneously with a conventional antipsychotic, were generally larger than necessary. These trials were commercially funded by the manufacturer of the atypical antipsychotic drugs. Only investigator-initiated trials were too small. The majority of large trials reported a statistically significant treatment effect, despite lack of clinical relevance, which confirms the presence of large sample size fallacy. The mean sample size was also higher when the study lasted longer than 6 weeks and was commercially funded, but this might be explained by the fact that placebo-controlled trials of atypical antipsychotics were generally longer and often industry-initiated. The chance of statistically significant findings was further enhanced by the use of multiple comparisons per study and multiple measurement scales per outcome in a number of the larger trials.

Many placebo-controlled trials of conventional antipsychotics had small sample sizes. Most were relatively old (published before 1990) and seemed to be investigator-initiated. Some of these trials reported clinically relevant results, but most were not statistically significant. That small placebo-controlled trials yielded statistically significant and clinically relevant effects relatively often might reflect publication bias.

Head-to-head trials had sample sizes that were (much) smaller than required, and these studies yielded non-statistically significant results that sometimes suggested a substantial effect. Even if we had set the limit for non-inferiority at 15%, the required sample would have been a lot higher than the sample sizes of the included studies were (346 without loss, and 385 with loss). It is unclear why these trials were so clearly underpowered. Perhaps, industry has little to gain from properly testing their own product against that of competitors. Non-commercial funds might not be interested in a trial with at least 2 × 433 patients to show that the tested drugs are non-inferior, even if patients might be quite willing to participate in a study that ensure treatment with an active drug.

### Sample Size Requirements

It is generally agreed that a trial protocol and report should report a sample size calculation (CONSORT Group, 2010). Nevertheless, only a third of trials in our review reported a sample size calculation and just three were complete. Although some trials can be considered old, most were published in the 1990s or later when it had become common to report trial methods in detail. Sample size calculations are often not (completely) reported in randomized trials in other fields of research was well (Chan et al., 2008; Charles et al., 2009). One review found that articles about newer randomized controlled trials included sample size calculations more often, and showed positive results more often (76%) than older studies (55%) (Latif et al., 2011).

Some studies in our review reported a lower alpha (2.5%) or beta (5%) than is usual in sample size calculations (5% and 20% respectively). In addition, the MCID proposed in the sample size calculations seemed rather small: difference in response rates <25% in 3/6 trials, and in relative risk reduction of <20% in 4/7 trials. The lower the alpha, beta, and MCID, the higher the calculated sample size will be and hence the power to detect a statistically significant but not clinically relevant treatment effect. Moreover, even if the expected difference is equal to the MCID, a proportion of the patients will not have a clinically relevant effect on the individual level. On the other hand, the expected drop-out rate in the sample size calculations was mostly lower than the (mean) reported drop-out, and this would have led to a spuriously smaller calculated sample size. Real drop-out might have been high because trial duration was long on average. Most trials lasted more than a month, even though in clinical practice, antipsychotics usually show an effect within 2 weeks, four at the most. It has been estimated that up to 64% of trials with continuous outcomes are underpowered or overpowered because of imprecise input (Tavernier and Giraudeau, 2015).

### Strengths and Limitations

To our knowledge determinants of sample size in trials testing antipsychotics for NPSs in dementia have not been studied previously. Our study showed that sample size calculations in the reports of these trials were missing on a large scale as was the correct interpretation of effect size. A limitation of our study is its focus on antipsychotic trials in dementia, which might be perceived as a small field of research. In addition, the interpretation of our results is limited by the possible presence of multiple testing. Many trials used multiple comparisons of either different drugs, different dosages, multiple outcomes, and sometimes multiple measurement instruments per outcome. Such multiple testing might reinforce the large sample size fallacy.

With our study, we do not want to suggest that large sample sizes should be avoided. It is important for clinical practice that study results are precise. Moreover, large sample sizes are very useful for identification of adverse effects. Small trials should not be avoided either, as long as they are published irrespective of results and available for pooling in meta-analyses.

The implication of our study is that researchers need to be encouraged to report and consider effect sizes in line with p-values to avoid the large sample size fallacy. Journals should probably mention this in their author instructions.

## Conclusion

Placebo-controlled trials that tested atypical antipsychotics showed large sample size fallacy. Placebo-controlled trials of conventional antipsychotics and head-to-head trials had insufficient power to detect a real difference between the treatment groups. Sample size calculations in antipsychotic trials for dementia need to be reported adequately.

## Data Availability Statement

The datasets generated for this study are available on request to the corresponding author.

## Author Contributions

TH, SJ, and HL extracted the data. TH and HL searched and selected the trials, performed the data analysis, and drafted the manuscript. SJ and SZ critically reviewed the manuscript and suggested revisions. HL designed the study.

## Conflict of Interest

The authors declare that the research was conducted in the absence of any commercial or financial relationships that could be construed as a potential conflict of interest.
