# Supplementary material for: Large Sample Size Fallacy in Trials About Antipsychotics for Neuropsychiatric Symptoms in Dementia
Source: Front Pharmacol. 2020 Feb 21;10:1701. doi: 10.3389/fphar.2019.01701 (PMC7047221; doi:10.3389/fphar.2019.01701)
Supplement: Supplementary file 1 [file DataSheet_1.docx]

**Supplement**

**Search string**

**In title/ abstract:** chlorpromazine OR levomepromazine OR promazine OR acepromazine OR triflupromazine OR cyamemazine OR chlorproethazine OR dixyrazine OR fluphenazine OR perphenazine OR prochlorperazine OR thiopropazate OR trifluoperazine OR acetophenazine OR thioproperazine OR butaperazine OR perazine OR periciazine OR thioridazine OR mesoridazine OR pipotiazine OR haloperidol OR trifluperidol OR melperone OR moperone OR pipamperone OR bromperidol OR benperidol OR droperidol OR fluanisone OR oxypertine OR molindone OR sertindole OR ziprasidone OR lurasidone OR flupentixol OR clopenthixol OR chlorprothixene OR tiotixene OR zuclopenthixol OR fluspirilene OR pimozide OR penfluridol OR loxapine OR clozapine OR olanzapine OR quetiapine OR asenapine OR clotiapine OR sulpiride OR sultopride OR tiapride OR remoxipride OR amisulpride OR veralipride OR levosulpiride OR prothipendyl OR risperidone OR mosapramine OR zotepine OR aripiprazole OR paliperidone OR iloperidone OR cariprazine OR brexpiprazole OR pimavanserin

**AND**

**Anywhere:** trial

**AND**

**Anywhere:** dementia

**Supplementary Table 1 NPI-NH in included studies that used this instrument**

| **Study** | **Baseline NPI-NH** | **Change in placebo group** |
| --- | --- | --- |
| Street 2000 | 43 | 28 |
| DeDeyn 2004 | 33 | 20 |
| Deberdt 2005 | 42 | 22 |
| DeDeyn 2005 | 40 | 19 |
| Schneider 2006 | 37 | 9 |
| Mintzer 2007 | 41 | 16 |
| Zhong 2007 | 36 | 23 |
| Paleacu 2008 | 41 | 19 |
| Streim 2008 | 38 | 19 |
| **Mean** | **39** | **19** |
